# Allergy-associated acute coronary syndrome without anaphylaxis in a prospective observational study

**DOI:** 10.1038/s41598-026-38633-1

**Published:** 2026-03-07

**Authors:** Mari Amino, Shie Takizawa, Seiji Morita, Kanae Kitatani, Ayumi Sasaki, Atsuhiko Yagishita, Yoshihide Nakagawa, Yuji Ikari, Koichiro Yoshioka

**Affiliations:** 1 Department of Cardiovascular Medicine, Tokai University, Shimokasuya 143, Isehara, Kanagawa 259-1193 Japan; 2 Department of Emergency and Critical Care Medicine, Tokai University, Shimokasuya 143, Isehara, Kanagawa 259-1193 Japan; 3 Department of Life Science Support, Research Innovation Center, University Hospitals Sector, Tokai University, Shimokasuya 143, Isehara, Kanagawa 259-1193 Japan

**Keywords:** Acute coronary syndrome, Allergy, Autonomic nervous system, Histamine, IgE, Kounis syndrome, Cardiology, Diseases, Immunology, Medical research

## Abstract

Kounis syndrome is an acute coronary syndrome (ACS) triggered by allergic reactions via mast cell activation, classically accompanied by anaphylaxis. However, ACS associated with mild or subclinical allergic reactions may be underrecognized. We investigated immune markers and autonomic nervous system activity in patients with ACS and characterized allergy-associated ACS without anaphylactic symptoms. Among 375 patients treated for ACS (April 2022 to March 2024), 49 were prospectively enrolled. Patients were classified into non-allergy-associated ACS (ACS-NA, n = 32) and allergy-associated ACS (ACS-AL, n = 17) groups. ACS-AL was subdivided into the non-anaphylactic (ACS-AL(nonA), n = 11) and anaphylactic (ACS-AL(withA), n = 6) groups. Allergic status was comprehensively assessed. Serum IgE and plasma histamine were measured at ACS onset. Heart rate variability (HRV) parameters were analyzed using 24-h ambulatory monitoring within 1 week after percutaneous coronary intervention. The ACS-AL(nonA) and ACS-AL(withA) groups showed significantly higher IgE and histamine levels than the ACS-NA group (P < 0.05), with overlapping distributions. HRV analysis revealed elevated LF/HF ratios and reduced daytime HF in both groups, indicating sympathetic predominance. ACS-AL, even without anaphylaxis, involves immune activation and autonomic imbalance. Combined assessment of IgE, histamine, and HRV may improve the identification of this phenotype.

**Supplementary Information:**

The online version contains supplementary material available at 10.1038/s41598-026-38633-1.

## Introduction

Allergic diseases are increasing worldwide, and approximately 30–40% of the global population is estimated to have some form of allergic disorder^1^. Many allergic conditions, including bronchial asthma, atopic dermatitis, allergic gastroenteritis, and allergic interstitial nephritis, are chronic diseases, in which immune cell activation and basal release of inflammatory mediators persist even during clinically quiescent periods. Immunoglobulin E (IgE) is reportedly involved in the development of cardiovascular disease and the progression of atherosclerosis^2–4^, whereas histamine exerts diverse modulatory effects on the myocardium, coronary arteries, and the autonomic nervous system^5,6^. Accordingly, increasing attention has been directed toward the impact of chronic allergic inflammation on cardiovascular pathophysiology.

Kounis syndrome (KS) was first described by Kounis and Zavras in 1991 as a pathological condition linking acute allergic reactions with acute coronary syndrome (ACS)^7^. In its typical presentation, acute mast cell activation triggered by allergen exposure induces coronary vasospasm, platelet activation, and plaque destabilization, resulting in a severe form of allergy-associated ACS accompanied by anaphylactic symptoms^8^. Conversely, the extent to which mild or clinically inconspicuous allergic reactions contribute to the acute-phase pathophysiology of ACS has not been sufficiently investigated.

Several limitations in the diagnosis and evaluation of allergy may underlie this knowledge gap. Specifically, methods for assessing allergic history are not standardized, including variability in the quantification of past allergic conditions, reliance on medical records or discharge summaries, and documentation of prior therapeutic interventions. Additionally, the presence or absence of acute allergic symptoms and the temporal relationship between allergen exposure and symptom onset are often inadequately evaluated. Furthermore, biological markers of allergy, such as histamine and IgE, are rarely measured during the acute phase of ACS, complicating accurate interpretation of disease mechanisms.

Beyond these diagnostic challenges, chronic allergic inflammation has been reported to induce myocardial and sympathetic nervous system remodeling^9,10^. Heart rate variability (HRV) analysis provides indices of autonomic nervous system activities, in which parasympathetic (vagal) activity—reflected by high-frequency (HF) power—mediates anti-inflammatory and cardioprotective effects, whereas sympathetic overactivity—reflected by an elevated low-frequency/HF (LF/HF) ratio—is associated with arrhythmogenesis and exacerbation of inflammation^11,12^. Histamine can act on both central and peripheral pathways to influence vagal and sympathetic nervous system activity^13,14^; however, no clinical studies have comprehensively evaluated immune mediators and autonomic nervous system activity during the acute phase of ACS.

**Fig. 1:**
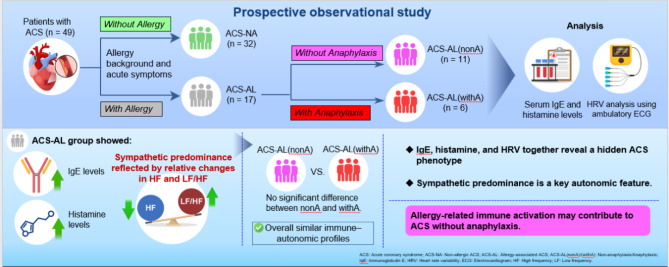
Overview of the study design, methodology, and main findings. Schematic representation summarizing the main observations of this study. In patients with allergy-associated acute coronary syndrome (ACS) without anaphylaxis, subclinical signs of autonomic imbalance characterized by relative sympathetic predominance were observed. Assessment of IgE, histamine, and heart rate variability may help identify such autonomic alterations. These findings are exploratory and intended to generate hypotheses for future studies and potential individualized management approaches. Image created by the authors in collaboration with Editage (www.editage.com).

Based on this background, we aimed to evaluate immune markers (IgE and histamine) and autonomic nervous system indices (HRV) in patients with ACS, and to clarify the characteristics of allergy-associated ACS in the absence of anaphylactic symptoms. The graphical abstract (Fig. 1) summarizes the pathogenic mechanisms discussed in this study.

## Results

### Comparison of patient characteristics

No significant differences in clinical backgrounds were observed between the non-allergy-associated ACS (ACS-NA) and allergy-associated ACS (ACS-AL) groups regarding age, sex, body mass index, coronary risk factors, laboratory findings at admission, left ventricular ejection fraction, electrocardiographic findings, culprit lesion location, medication use, or short-term outcomes (Table 1).

**Table 1:** Patient characteristics.

	ACS-NA (n = 32)	ACS-AL (n = 17)	P value	ACS-AL(nonA) (n = 11)	ACS-AL(withA) (n = 6)	P value
Age (years)	71 ± 13.4	69 ± 8.5	n.s	66 ± 9.2	75 ± 2.8	n.s
Men	25 (78.1%)	15 (88.2%)	n.s	9 (81.8%)	6 (100%)	n.s
BMI (kg/m^2^)	23.9 ± 4.0	23.6 ± 4.0	n.s	22.8 ± 4.2	24.3 ± 3.8	n.s
Coronary risk						
Hypertension	25 (78.1%)	13 (76.5%)	n.s	8 (72.7%)	5 (83.3.%)	n.s
Dyslipidemia	20 (62.5%)	11 (64.7%)	n.s	7 (63.6%)	4 (66.7%)	n.s
Diabetes Mellitus	10 (31.3%)	6 (35.3%)	n.s	4 (36.4%)	2 (33.3%)	n.s
Smoking	7 (21.9%)	4 (23.5%)	n.s	3 (27.3%)	1 (16.7%)	n.s
Obesity	4 (12.5%)	3 (17.6%)	n.s	1 (9.1%)	2 (33.3%)	n.s
Laboratory data						
WBC (× 10^9^/L)	9.5 ± 4.7	10.2 ± 4.2	n.s	11.2 ± 4.6	8.4 ± 3.0	n.s
Eosinophils (%)	1.9 ± 2.1	2.0 ± 2.1	n.s	1.7 ± 2.4	2.2 ± 1.3	n.s
Basophils (%)	0.4 ± 0.3	0.5 ± 0.4	n.s	0.4 ± 0.4	0.6 ± 0.5	n.s
CPK (U/L)	532 ± 741.7	658 ± 848.5	n.s	666 ± 1016.1	643 ± 487.9	n.s
AST (U/L)	101 ± 145.2	80 ± 91.1	n.s	77 ± 114.7	87 ± 12.5	n.s
ALT (U/L)	45 ± 59.7	36 ± 22.9	n.s	32 ± 23.1	45 ± 22.0	n.s
LDH (U/L)	402 ± 208.8	421 ± 416.9	n.s	432 ± 475.0	401 ± 322.8	n.s
Cr (mg/dl)	1.8 ± 2.7	1.5 ± 1.5	n.s	1.6 ± 1.9	1.1 ± 1.2	n.s
BUN (mg/dl)	21.9 ± 13.8	20.4 ± 10.8	n.s	21.0 ± 13.3	19.2 ± 4.2	n.s
CRP (mg/dl)	2.9 ± 4.1	4.4 ± 6.1	n.s	4.9 ± 7.6	3.6 ± 3.1	n.s
Troponin-T (ng/mL)	813 ± 1249.2	919 ± 1209.7	n.s	1019.5 ± 1326.4	741 ± 1011.3	n.s
BNP (pg/ml)	340 ± 479.4	481 ± 1081.9	n.s	579 ± 1327.4	301 ± 401.8	n.s
LVEF (%)	52 ± 13.1	53 ± 10.1	n.s	51 ± 9.5	57 ± 11.1	n.s
ECG diagnosis						
Anterior	16 (50.0%)	6 (35.3%)	n.s	5 (45.5%)	1 (16.7%)	n.s
Lateral	4 (12.5%)	2 (11.8%)	n.s	1 (9.1%)	1 (16.7%)	n.s
Inferior	8 (25.0%)	6 (35.3%)	n.s	4 (36.4%)	2 (33.3%)	n.s
Posterior	0	0	n.s	0	0	n.s
Non-ST elevation	4 (12.5%)	3 (17.6%)	n.s	1 (9.1%)	2 (33.3%)	n.s
Culprit lesion						
LAD	15 (46.9%)	6 (35.3%)	n.s	4 (36.4%)	2 (33.3%)	n.s
LCx	3 (9.3%)	2 (11.8%)	n.s	2 (18.2%)	0	n.s
RCA	7 (21.9%)	4 (23.5%)	n.s	2 (18.2%)	2 (33.3%)	n.s
Multiples	5 (15.6%)	2 (11.8%)	n.s	1 (9.1%)	1 (16.7%)	n.s
VSA	2 (6.3%)	3 (17.6%)	n.s	2 (18.2%)	1 (16.7%)	n.s
Medications*						
Anti-platelet	32 (100%)	17 (100%)	n.s	11 (100%)	6 (100%)	n.s
Statin	31 (96.9%)	17 (100%)	n.s	11 (100%)	6 (100%)	n.s
Beta-blocker	30 (93.8%)	14 (82.4%)	n.s	11 (100%)	3 (50.0%)	n.s
Nitrates	12 (37.5%)	6 (35.3%)	n.s	3 (27.3%)	3 (50.0%)	n.s
CCB	9 (28.1%)	5 (29.4%)	n.s	2 (18.2%)	3 (50.0%)	n.s
AAD	3 (9.4%)	1 (5.9%)	n.s	1 (9.1%)	0	n.s
Prognosis						
Death	3 (9.4%)	2 (11.8%)	n.s	2 (18.2%)	0	n.s
Hospital transfer	15 (46.9%)	6 (35.3%)	n.s	5 (45.5%)	1 (16.7%)	n.s
Discharge to home	14 (43.8%)	9 (52.9%)	n.s	4 (36.4%)	5 (83.3%)	n.s

ACS, acute coronary syndrome; ACS-NA, Non-allergic ACS; ACS-AL, Allergy associated ACS; ACS-AL(nonA), ACS- allergy without anaphylaxis; ACS-AL(withA), ACS- allergy with anaphylaxis; BMI, Body mass index; WBC, white blood cell; CPK, creatine phosphokinase; AST, Aspartate aminotransferase; ALT, Alanine transaminase; LDH, lactate dehydrogenase; Cr, Creatinine; BUN, Urea nitrogen; CRP, C-reactive protein; BNP, Brain natriuretic peptide; LVEF, Left ventricular ejection fraction; ECG, Electrocardiogram; LAD, Left Anterior Descending artery; LCx, Left Circumflex artery; RCA, Right Coronary Artery; VSA, vasospastic angina**;** CCB, Calcium channel blockers; AAD, Antiarrhythmic drugs; *Medications, Post-PCI prescriptions; Data are presented as mean ± standard deviation. Nominal scales in each group are shown as a number (%). Statistics were performed by comparing the two groups, ACS-NA vs. ACS-AL, ACS-AL(nonA) vs. ACS-AL(withA), with a significance level of p < 0.05, “n.s” indicates p ≥ 0.05.

When the ACS-AL group was subdivided into the non-anaphylactic and anaphylactic groups—namely the ACS-AL(nonA) and ACS-AL(withA) groups, respectively—the rate of β-blocker use after percutaneous coronary intervention (PCI) was lower in the latter group (100% vs. 50%). This difference likely reflects clinical concern regarding reduced responsiveness to epinephrine during anaphylaxis, which potentially discouraged β-blocker initiation in patients in the ACS-AL(withA) group^15^.

Detailed allergy-related information is summarized in Table 2. No difference was found in allergy history (AH) scores between the ACS-AL(nonA) and ACS-AL(withA) groups. Chart documentation for patients with AH scores of 3 to 4 was as follows: in the ACS-AL(nonA) group, IgE records were available in 9/9 patients, radioallergosorbent test (RAST) records in 6/9, and skin test records in 3/9 (with overlap). In the ACS-AL(withA) group, IgE records were available in 4/4 patients, RAST records in 4/4, and skin test records in 1/4 (with overlap).

**Table 2:** Detailed Allergy information.

	ACS-AL(nonA) (n = 11)	ACS-AL(withA) (n = 6)	P value
Allergy-History (AH) score			
AH score 0	0	0	n.s
AH score 1	1 (9.1%)	0	n.s
AH score 2	1 (9.1%)	2 (33.3%)	n.s
AH score 3	8 (72.7%)	4 (66.7%)	n.s
AH score 4	1 (9.1%)	0	n.s
Allergy-Severity (AS) grade			
AS grade 0	0	0	n.s
AS grade 1	11 (100%)	0	< 0.01
AS grade 2	0	6 (100%)	< 0.01
Time-Relation (TR) grade			
TR grade 0	1 (9.1%)	0	n.s
TR grade 1	5 (45.5%)	0	n.s
TR grade 2	5 (45.5%)	6 (100%)	0.04
Time-1 from exposure (min)	300.0 (55–330)	11.0 (5.5–19.5)	< 0.01
Time-2 from exposure (min)	480.0 (70–1260)	37.5 (22.3–40.0)	< 0.01
Allergen in this instance*			
Contrast agents	2 (18.2%)	1 (16.7%)	n.s
Pollen	2 (18.2%)	1 (16.7%)	n.s
Bees	0	3 (50.0%)	0.03
Bronchial asthma	2 (18.2%)	0	n.s
Antibiotics	2 (18.2%)	0	n.s
Sedatives	0	1 (16.7%)	n.s
PPI	1 (9.1%)	0	n.s
COVID19-Vaccine	1 (9.1%)	0	n.s
Taro	1 (9.1%)	0	n.s
Tuna	0	1 (16.7%)	n.s
Anisakis	0	1 (16.7%)	n.s
Trigger undetermined	1 (9.1%)	0	n.s

ACS, acute coronary syndrome; ACS-NA, Non-allergic ACS; ACS-AL, Allergy associated ACS; ACS-AL(nonA), ACS- allergy without anaphylaxis; ACS-AL(withA), ACS- allergy with anaphylaxis; Time from exposure was defined as the interval between exposure and onset of allergic symptoms (Time-1) or ACS diagnosis (Time-2), and is presented as the median with interquartile range; Allergen, *Data include overlapping cases; PPI, proton pump inhibitor; Trigger undetermined: Allergic symptoms were present (wheezing, skin rash, and pruritus), but the causative allergen could not be identified (likely food-related), Nominal scales in each group are shown as a number (%). Statistics were performed by comparing the two groups, ACS-NA vs. ACS-AL, ACS-AL(nonA) vs. ACS-AL(withA), with a significance level of p < 0.05, “n.s” indicates p ≥ 0.05.

Regarding treatment history, patients in the ACS-AL(nonA) group had received antihistamines (8/9 patients), antileukotriene agents (3/9), and steroids (2/9) (with overlap). In the ACS-AL(withA) group, antihistamines were used in 4/4 patients, antileukotriene agents in 1/4, and steroids in 0/4. The duration of allergic disease was 11.4 years (interquartile range [IQR]: 3 to 21 years) in the ACS-AL(nonA) group and 4.7 years (IQR: 2 to 6 years) in the ACS-AL(withA) group.

All patients in the ACS-AL(nonA) group met Allergy Severity (AS) grade 1 criteria, whereas all patients in the ACS-AL(withA) group met AS grade 2 criteria, consistent with the predefined classification. No patient exhibited documented acute exacerbation of chronic allergic disease at the time of ACS onset.

Regarding Time-Relation (TR) grade, in the ACS-AL(nonA) group, 46% of cases occurred within 4 h of exposure, and 46% occurred between 4 and 24 h, indicating delayed onset following allergic reactions. Conversely, the ACS-AL(withA) group showed a median exposure-to-onset time of 11 min, confirming a close temporal association with acute onset. As TR was not a mandatory criterion in the ACS-AL(nonA) category, heterogeneity and undetermined timing were present, whereas timing was documented at the minute level in the ACS-AL(withA) group.

Identified allergens in the ACS-AL group included contrast media, pollen, bee venom, medications, foods, and vaccines; only one case in the ACS-AL(nonA) group had an undetermined trigger. However, because this patient exhibited acute symptoms corresponding to AS grade 1 and had an AH score ≥ 1, the case was classified as ACS-AL(nonA) with TR grade 0. In the ACS-AL(withA) group, bee venom was the most frequent trigger; all six patients developed cutaneous, as well as respiratory or circulatory symptoms, requested emergency transport, and were diagnosed with ACS within 30 min of hospital arrival.

### IgE and histamine

**Fig. 2:**
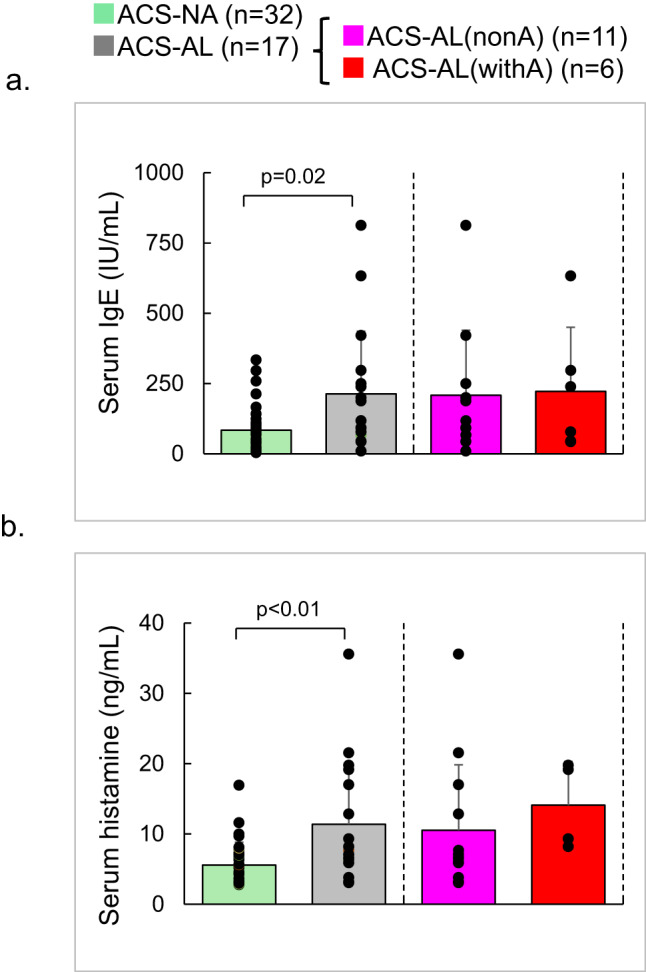
Blood IgE and histamine concentrations. (**a**) Serum IgE concentrations were higher in the ACS-AL group than in the ACS-NA group (213.6 vs. 84.5 IU/mL, P = 0.02). However, interindividual variability was large, and values in both groups spanned both below and above the reference range. No significant difference was observed between the ACS-AL(nonA) and ACS-AL(withA) groups. (**b**) Plasma histamine concentrations were also higher in the ACS-AL group (11.4 vs. 5.6 ng/mL, P < 0.01). No significant difference was observed between the ACS-AL(nonA) and ACS-AL(withA) groups.

Serum IgE levels were significantly higher in the ACS-AL group than in the ACS-NA group (213.6 ± 221.9 vs. 84.5 ± 73.7 IU/mL, P = 0.02; Fig. 2a). However, interindividual variability was substantial, and values in both groups overlapped the reference upper limit of 170 IU/mL. No significant difference was observed between the ACS-AL(nonA) and ACS-AL(withA) groups.

Plasma histamine levels were also higher in the ACS-AL group than in the ACS-NA group (11.4 ± 8.7 vs. 5.6 ± 3.2 ng/mL, P < 0.01; Fig. 2b). Similarly, no significant difference was observed between the ACS-AL(nonA) and ACS-AL(withA) groups. In four healthy adult volunteers without a history of allergy, plasma histamine levels were 3.7 ± 2.9 ng/mL (not shown in Fig. 2b).

### HRV analysis

**Fig. 3:**
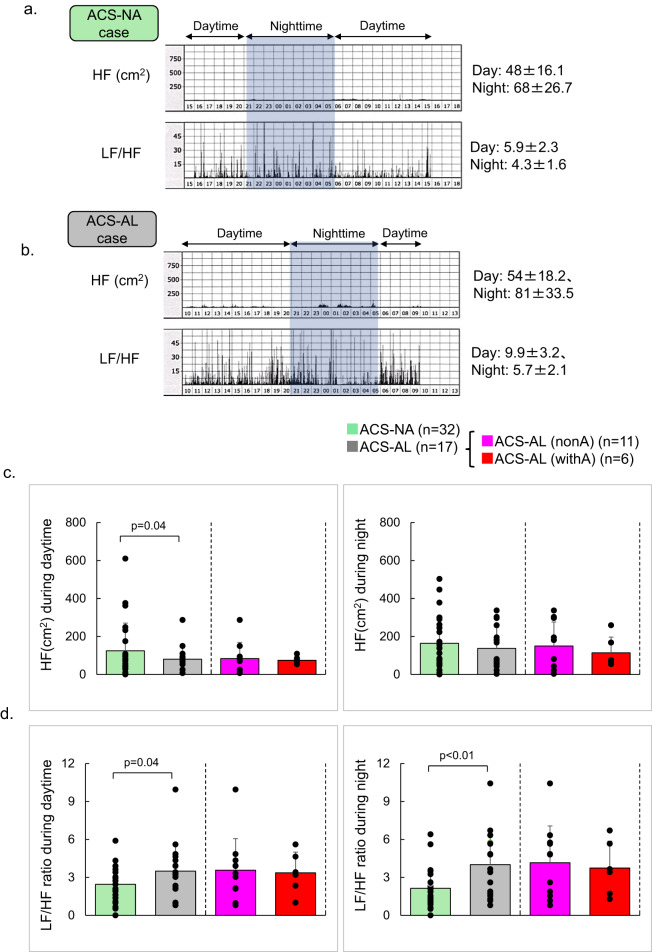
Sympathetic activation and disrupted circadian rhythm. (**a**) Representative recordings from a patient in the ACS-NA group. High-frequency (HF) power, an index of parasympathetic activity, was markedly suppressed throughout the day, and the low-frequency to high-frequency (LF/HF) ratio remained relatively high even at night (patient receiving β-blockers), resulting in a loss of the normal circadian rhythm. (**b**) Representative recordings from a patient in the ACS-AL group. HF power was suppressed similarly to the ACS-NA patient, whereas the LF/HF ratio increased further, suggesting enhanced sympathetic activity (patient receiving β-blockers). (**c**) In the overall analysis, daytime HF power was lower in the ACS-AL group than in the ACS-NA group, whereas no significant difference was observed during the nighttime. No significant difference was observed between the ACS-AL(nonA) and ACS-AL(withA) groups. (**d**) The LF/HF ratio was higher in the ACS-AL group throughout the day, suggesting relative sympathetic predominance. No significant difference was observed between the ACS-AL(nonA) and ACS-AL(withA) groups.

Representative HRV recordings from patients in the ACS-NA and ACS-AL groups are shown in Fig. 3. In the ACS-NA example, HF tone, reflecting parasympathetic activity, was extremely low from daytime through sleep, with near-complete loss of circadian variation (Fig. 3a). Consequently, the LF/HF ratio was relatively elevated during both daytime and nighttime.

In the ACS-AL example, HF tone was similarly low throughout the day with minimal circadian fluctuation (Fig. 3b). However, LF/HF values were consistently higher than those observed in the ACS-NA example.

In the pooled analysis, daytime HF was significantly lower in the ACS-AL group than in the ACS-NA group, whereas no significant difference was observed during nighttime (Fig. 3c). Conversely, the LF/HF ratio was significantly higher in the ACS-AL group throughout the day, indicating relative sympathetic predominance (daytime: 3.5 ± 2.2 vs. 2.5 ± 1.3, P = 0.04, 95% confidence interval [CI], − 0.2 to + 2.2, Welch df = 22.1; nighttime: 4.0 ± 2.6 vs. 2.1 ± 1.5, P < 0.01, 95% CI, + 0.5 to + 3.3, Welch df = 21.8) (Fig. 3d).

Effect sizes (Cohen’s d) exceeded 0.8 in all comparisons, indicating large between-group differences and clinically meaningful autonomic changes. Notably, nighttime sympathetic predominance remained robust despite the limited sample size. No significant differences were observed between the ACS-AL(nonA) and ACS-AL(withA) groups. Medications that could influence autonomic indices, including β-blockers, were continued based on clinical judgment; therefore, results should be interpreted with caution.

### Arrhythmia treatment in ACS-AL(nonA)

**Fig. 4:**
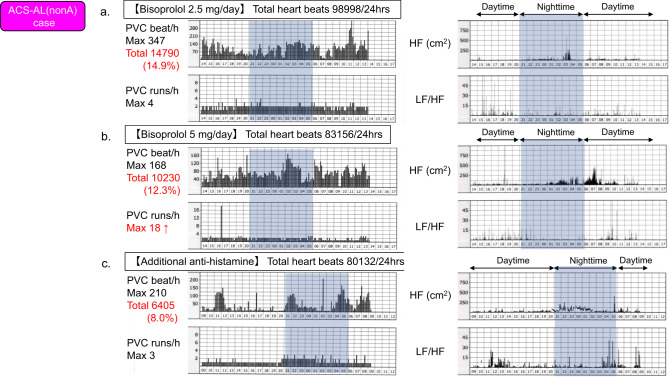
Clinical course of treatment for premature ventricular contractions (PVCs). (**a**) In a representative case involving a patient in the ACS-AL(nonA) group, frequent PVCs (14.9%) were observed despite β-blocker therapy (bisoprolol 2.5 mg). HRV analysis showed marked suppression of HF power and an elevated LF/HF ratio throughout the day. (**b**) After the bisoprolol dose was increased to 5 mg, the PVC burden decreased to 12.3%, but the number of PVC runs increased. HRV analysis showed an increase in HF power, with a tendency for HF power to remain elevated after waking, indicating a disrupted circadian rhythm. (**c**) After the addition of an antihistamine, the PVC burden decreased to 8.0%, and PVC runs were suppressed. HRV analysis showed a nighttime increase in HF power and an increase in the LF/HF ratio just before waking, suggesting a trend toward normalization of the circadian rhythm.

In one patient in the ACS-AL(nonA) group, premature ventricular contractions (PVCs) accounted for 14.9% of total heartbeats, with a limited response to β-blocker therapy (bisoprolol 2.5 mg/d) (Fig. 4a, left). Increasing the bisoprolol dose to 5 mg/d resulted in persistent PVCs (12.3%) and an increased number of PVC runs (maximum of 18 beats) (Fig. 4b, left). After the addition of an antihistamine, the PVC burden decreased to 8.0%, and PVC runs were reduced to three episodes (Fig. 4c, left).

HRV analysis revealed that, compared with the first and second measurements, the third recording showed an increase in HF beginning at nighttime, suggesting partial restoration of the circadian rhythm (Fig. 4a–c, right). Although this represents a single case, we hypothesize that this observation supports a potential link between histamine signaling and vagal modulation. This case is presented solely for conceptual illustration and does not imply causality.

## Discussion

In this study, we prospectively collected cases of ACS accompanied by allergy or anaphylaxis and simultaneously evaluated IgE, histamine, and HRV indices. We found that allergy-associated ACS was characterized by significantly enhanced sympathetic activity compared with ACS without allergy. In the allergy-associated group, IgE and histamine levels were elevated and sympathetic predominance persisted regardless of the apparent severity of allergy. These findings suggest that histamine influences acute inflammation and autonomic activity in ACS even when allergic symptoms are mild or exposure is not clearly identified.

KS broadly encompasses allergy-associated ACS; however, discussion has primarily focused on anaphylaxis, a severe allergic manifestation. In contrast, the extent to which chronic allergic predisposition influences acute-phase ACS pathophysiology has remained unclear. In the present study, the ACS-AL(nonA) group showed elevated histamine levels and sympathetic predominance despite relatively non-severe acute symptoms. Given that allergic reactions are heterogeneous and chronic or latent immune activation may contribute, temporal proximity was not a mandatory requirement for defining non-anaphylactic allergy-associated ACS; nevertheless, allergen exposure within 24 h was confirmed observationally in most ACS-AL(nonA) cases.

Although causal inference cannot be considered definitive, these results suggest that within the population previously regarded as having “ACS unrelated to allergy,” a subgroup exists that is characterized by autonomic changes driven by excessive immune activation. In modern society, more than one-third of the population has some form of allergy, which may serve as a potential trigger for ACS. If allergen exposure occurs repeatedly without recognition, it could increase not only the risk of ACS onset but also the risk of recurrence after treatment; therefore, early identification of this pathophysiology and appropriate intervention are essential.

IgE is not only a marker of allergen sensitization^2^ but also has been implicated in atherosclerosis progression and plaque destabilization^3^. IgE-dependent mast cell activation promotes endothelial injury through the release of tumor necrosis factor-α, IL-6, proteases, and lipid mediators, thereby contributing to the progression of coronary lesions^8^. Histamine reflects ongoing allergic reactions and can induce acute changes in coronary plaque instability and myocardial contractility^16,17^. Because histamine is unstable in the blood, has a short half-life, and is difficult to handle in routine testing, the development of simpler and more broadly applicable measurement methods is desirable. Both IgE and histamine levels were higher in the ACS-AL(nonA) and ACS-AL(withA) groups than in the ACS-NA group, suggesting their potential as adjunct biomarkers of allergy-associated inflammation. Establishing systems to enable future clinical implementation would be beneficial.

While histamine-mediated heart rate regulation via H₁ receptors appears limited, H₂ receptors are involved in parasympathetic activity, and their blockade has been reported to induce sympathetic predominance^13^. In addition, H₃ receptors regulate acetylcholine release from vagal terminals and may contribute to heart rate control^14^. Thus, histamine may modulate parasympathetic activity depending on the receptor subtype and site of action, but its effects in the acute phase of ACS may be modified in a disease-dependent manner. In our HRV analysis, HF power was reduced regardless of allergy status, suggesting that the profound suppression of vagal activity associated with the physiological stress of ACS^11^ potentially masked histamine-related modulatory effects.

In contrast, the LF/HF ratio, an index of relative sympathetic activity, was higher in the ACS-AL group. This may reflect increased sympathetic excitability in response to vagal suppression during the acute phase of ACS^18^. Another possible explanation for the greater elevation compared with the ACS-NA group is that the persistent release of histamine and cytokines from chronic mast cell activation may accelerate neurodegeneration driven by microinflammation^10,19^. These inflammatory mediators may act as an immune response superimposed on the general inflammatory reaction associated with ACS, promoting myocardial and neural remodeling^20^ and potentially leading to autonomic imbalance through the excessive sprouting of immature sympathetic nerves. Indeed, as shown in Fig. 4, one illustrative case demonstrated a nighttime increase in HF power after the addition of an antihistamine when β-blockade alone appeared insufficient, suggesting a possible relationship. Autonomic modulation related to allergy may be superimposed on the sympathetic activation inherent to ACS.

In our comparison of the ACS-AL(nonA) and ACS-AL(withA) groups, tryptase was not measured; therefore, direct evidence of acute mast cell activation was not provided^21^. Nonetheless, in the ACS-AL(withA) group, the rapidly progressive course within minutes to 4 h after allergen exposure was clear (Table 2), consistent with the typical pathophysiological concept of KS. In the ACS-AL(withA) group, rapid systemic responses triggered by allergen exposure may markedly activate mast cells in the myocardium and the coronary culprit lesion, leading to the production of large amounts of histamine and inflammatory mediators. The release of histamine into the systemic circulation causes peripheral vasodilation and reduces venous return, which potentially induces strong sympathetic activation through baroreceptor reflexes^6,22^.

Compared with the ACS-AL(withA) group, the ACS-AL(nonA) group includes cases with less clearly defined symptom onset, and these two subtypes have not traditionally been discussed within the same category. However, because the histamine and HRV results partly overlap with those in the ACS-AL(withA) group, our findings suggest that these entities may be viewed within a continuous disease spectrum. The reported diagnostic rate of KS ranges from 1.1%^23^ to 3.4%^24^, yet awareness in clinical practice remains low, and the true incidence may be higher. In 2021, the Japanese Ministry of Health, Labour and Welfare issued a safety alert regarding the risk of drug-induced KS, given the severity of allergy-associated cardiac disease^25^. Although no international guidelines for diagnosis and treatment currently exist, the presence of ACS-AL(nonA) may prompt a reconsideration of ACS pathophysiological classification and risk stratification.

This study has some limitations. First, this was a single-center, small-scale exploratory observational study with a limited sample size. In particular, the number of ACS-AL(withA) cases was small; therefore, we determined that multivariable adjustment and sensitivity analyses, including confounders, presented a high risk of unstable estimates and overfitting, and these analyses were not performed. Accordingly, the findings should be interpreted as associations rather than established causal inferences. Validation in multicenter, large-scale studies is required for generalization. Second, we used medications that affect autonomic function, such as β-blockers and calcium channel blockers, as shown in Table 1. Although adjustment was not performed because of the small sample size, multiple potential factors may influence the interpretation of the results, including the sleep environment and psychological stress. In addition, the use of the LF/HF ratio as an index of sympathetic predominance remains a matter of debate. Third, the serum tryptase level was not measured. Although tryptase would be a desirable supportive diagnostic marker for KS, it requires blood sampling within 1–2 h after onset and rapid processing, which poses substantial operational constraints in emergency ACS care. Future studies should evaluate biomarkers, including tryptase, under standardized conditions in combination with histamine.

In conclusion, this study compared the immunological and autonomic characteristics of allergy-associated ACS (ACS-AL) with those of non-allergic ACS (ACS-NA). Even when allergic symptoms were mild, elevated IgE and histamine levels and increased sympathetic activity were observed, suggesting that these conditions may be reconsidered within a continuous disease spectrum of KS. This study provides exploratory findings to support further investigation into the involvement of allergy in patients with ACS.

## Methods

### Study design and population

**Fig. 5:**
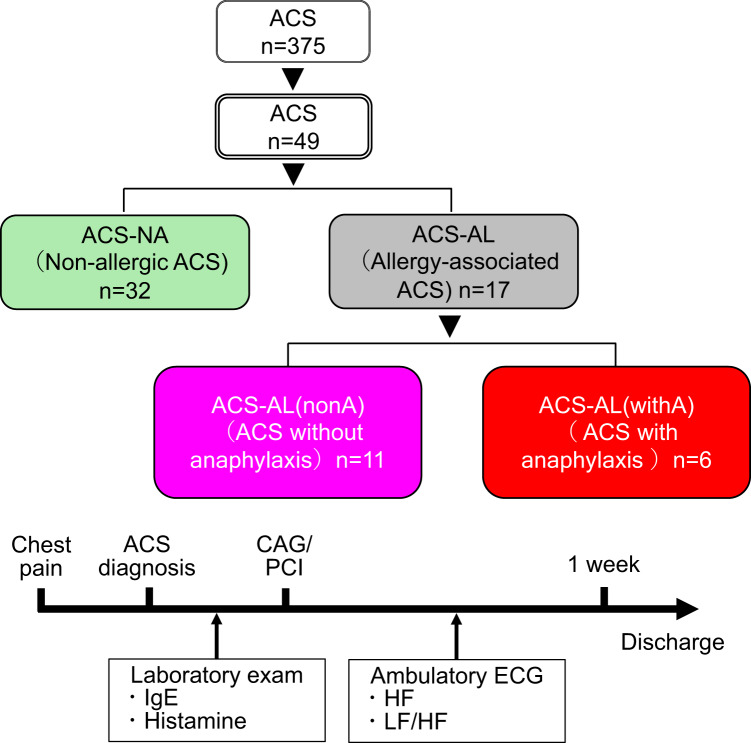
Classification of patients based on allergic history. Patients with acute coronary syndrome (ACS) were classified into a non-allergic group (ACS-NA; green) and an allergy-associated group (ACS-AL; gray) based on a history of allergic disease. The ACS-AL group was further subdivided into the ACS-AL(nonA) (pink) and ACS-AL(withA) (red) subgroups. Blood samples were collected after the diagnosis of ACS to measure IgE and histamine levels. Within 1 week after PCI, 24-h ambulatory ECG monitoring was performed; parasympathetic activity (HF power) and sympathovagal balance (LF/HF ratio) were assessed using frequency-domain analysis under β-blocker therapy. Analyses were conducted separately for daytime and nighttime periods and cross-checked against recorded sleep–wake times.

This prospective observational study enrolled consecutive patients diagnosed with ACS at our institution between April 2022 and March 2024. The study was approved by the Institutional Review Board of Tokai University (21R224), and all participants provided written informed consent. Of the 375 patients diagnosed with ACS during the study period, 49 patients who provided consent and from whom blood samples and electrocardiographic (ECG) data were obtained prior to therapeutic intervention were included in the final analysis (Fig. 5). The diagnosis of ACS was established in accordance with the guidelines of the Japanese Circulation Society, based on ischemic symptoms such as chest pain, ECG changes, and the elevation of cardiac biomarkers.

### Assessment of AH and exposures

First, AH was systematically assessed using multiple information sources: structured patient interviews (addressing allergic rhinitis, pollinosis, bronchial asthma, drug allergy, and food allergy), medical record documentation, medication and adverse reaction history, diagnoses made by allergists or attending physicians (including IgE testing, RAST, and skin test records), and allergy-related treatment history (including use of anti-allergic medications, epinephrine administration, hospitalization, and duration of treatment).

Based on these data, AH was quantified using a 0–4-point scoring system (AH score):

- AH 0: no history of allergy;
- AH 1: self-reported allergic symptoms without treatment;
- AH 2: mild allergic symptoms documented in medical records without treatment;
- AH 3: physician-diagnosed allergy with therapeutic intervention;
- AH 4: history of hospitalization due to allergic disease.

Second, acute allergic symptoms at the time of ACS diagnosis were assessed via structured questioning regarding sneezing, rhinorrhea, urticaria, pruritus, flushing, nausea, diarrhea, throat discomfort, dyspnea, wheezing, angioedema, impaired consciousness, and hypotension. These symptoms were classified using an AS Grade:

AS 0: no symptoms;

AS 1: localized mild allergic symptoms;

AS 2: systemic and rapidly progressive severe allergic symptoms involving multiple organs (e.g., cutaneous, respiratory, and circulatory systems).

Third, allergen exposure within 48 h before the onset of ACS (including medications, foods, insect stings, and environmental factors) was assessed, and the temporal relationship between exposure and symptom onset was classified into three TR grades:

TR 0: onset ≥ 24 h after exposure or exposure timing unknown;

TR 1: onset within 4–24 h after exposure;

TR 2: onset within 4 h after exposure.

Given the challenges of precise records of exposure timing in emergency clinical settings, “within several hours” was operationally defined as TR 2.

In ACS-AL(nonA), temporal proximity (TR grade) was not a mandatory diagnostic criterion, based on the hypothesis that chronic or subclinical allergic immune activation influences autonomic nervous system activity and inflammatory status independently of immediate allergen exposure.

Based on the combined evaluation of these three components, patients were classified into the ACS-NA (n = 32) and ACS-AL (n = 17) groups. The ACS-AL group was further subdivided based on the presence of anaphylaxis into the ACS-AL(nonA) (n = 11) and ACS-AL(withA) (n = 6) subgroups. The classification criteria were as follows:

1. **ACS-NA:** Absence of AH (AH score = 0), absence of acute allergic symptoms (AS 0), and minimal or no identifiable allergen exposure.
2. **ACS-AL(nonA):** Presence of AH (AH score ≥ 1), presence of acute allergic symptoms (AS 1), and no requirement for temporal proximity between allergen exposure and symptom onset (TR grade 0 permitted). This group was defined as an exploratory category emphasizing clinical background and acute symptoms without requiring definitive evidence of immediate-type allergic reactions at ACS onset.
3. **ACS-AL(withA):** Presence of AH (AH score ≥ 1), presence of acute anaphylactic symptoms (AS 2), and a clear temporal relationship between allergen exposure and symptom onset (TR grade ≥ 1 required).

### Blood sampling and biomarker measurement

Serum IgE and plasma histamine levels were not used for group classification but were analyzed as biological markers independent of the diagnostic definitions. Blood samples were obtained upon emergency department admission following the onset of ACS, and total IgE and histamine levels were measured. Samples were promptly centrifuged, and plasma was stored frozen. Routine laboratory tests and total IgE measurements were performed immediately after sampling.

Because histamine has a short half-life and is highly sensitive to storage conditions, it is not routinely used in standard clinical testing. In this study, plasma histamine levels were measured using a validated liquid chromatography–tandem mass spectrometry method previously established domestically^26^. As a reference, plasma histamine levels were measured in four age-matched healthy adults without a history of allergy (with prior approval from the institutional ethics committee).

### Ambulatory electrocardiographic recording

In patients with ACS, 24-h ambulatory ECG monitoring was performed within 1 week after percutaneous coronary intervention (mean, 4.8 ± 1.1 day). Recordings containing more than 10% artifacts were repeated. Frequency-domain HRV analysis was conducted to calculate HF power as an index of parasympathetic activity and the LF/HF ratio as an index of sympathovagal balance.

Based on scheduled ward lights-off times, recordings were divided into daytime (06:00–21:00) and nighttime (21:00–06:00) periods. Sleep and wake times were verified using activity logs; mean values for each period were used for analysis. Medications affecting heart rate, including β-blockers, were continued during HRV recording.

### Statistical analysis

Categorical variables were compared between groups using the chi-square test or Fisher’s exact test, as appropriate. Continuous variables are expressed as mean ± standard deviation, and between-group comparisons were performed using Welch’s t-test to account for differences in sample size and variance. All statistical tests were two-tailed, with a significance threshold of P < 0.05.

Effect sizes were evaluated using Cohen’s d: values < 0.2 were considered small; approximately 0.5, moderate; and ≥ 0.8, large. For primary outcomes (e.g., LF/HF and HF), mean differences between groups and 95% confidence intervals were calculated based on Welch’s t-test. Given the exploratory nature of this study, no correction for multiple comparisons was applied; results were interpreted with an emphasis on effect sizes and confidence intervals. Statistical analyses were performed using SPSS version 30 (IBM Corp., Armonk, NY, USA). No missing data were present in the analyzed variables.

## Supplementary Information

Supplementary Information.

## Data Availability

The datasets presented in this article are not readily available because The data cannot be shared due to institutional data ownership and privacy concerns. Given the small number of participants and the sensitive nature of the clinical information, there is a potential risk of re-identification, even with anonymization. Requests to access the datasets should be directed to Mari Amino, mariamino@tokai.ac.jp.

## Declarations

### AI Use

Generative AI tools were used for language editing and stylistic refinement of the manuscript. The authors take full responsibility for the content, interpretation, and conclusions presented, and no AI tools were used for data analysis or generation of scientific content.
