# Supplementary material for: Allergy-associated acute coronary syndrome without anaphylaxis in a prospective observational study
Source: Sci Rep. 2026 Mar 7;16:12491. doi: 10.1038/s41598-026-38633-1 (PMC13087291; doi:10.1038/s41598-026-38633-1)
Supplement: Supplementary file 1 — Supplementary Information. [file 41598_2026_38633_MOESM1_ESM.docx]

**Supplementary data**

**Methods**

1. **Histamine calculation**
   1. **Chemicals and reagents**
      Histamine dihydrochloride was purchased from FUJIFILM Wako Pure Chemical Corporation (Osaka, Japan). The internal standard (IS), d4-histamine was purchased from Cambridge Isotope Laboratories, Inc. (Tewksbury, MA, USA). Acetonitrile was sourced from Honeywell (NC, USA). Methanol, ultrapure water (UPW), formic acid (FA), and ammonium bicarbonate were also obtained from Fujifilm (Tokyo, Japan). A 2% ammonium methanol solution was purchased from Kanto Kagaku (Tokyo, Japan).
   2. **Standard solutions for histamine analysis**
      Stock solutions of histamine (1 µM) and d4-histamine (1 µM) in UPW were prepared by dilution prior to each analysis and stored at –80°C. Calibration standard solutions of histamine (0, 0.1, 1.0, 10, 20, 50, 100 pmol) were prepared by diluting the stock solution with 0.1% FA in UPW. D4-histamine (200 pmol) was diluted with UPW and spiked into each standard solution and sample prior to LC-MS/MS injection.
   3. **Sample preparation**
      To 100 µl of plasma, 200 pmol of d4-histamine was added as an internal standard, followed by 900 µl of 5 mM ammonium bicarbonate buffer (pH 7.6). Solid-phase extraction was performed using an InertSep MCX 10 mg/1 ml cartridge (GL Sciences, Tokyo, Japan). The cartridge was conditioned with methanol and UPW, washed with UPW, and analytes were eluted with 1 ml of 2% ammonium methanol. After extraction, samples were dried using a SpeedVac (Thermo Fisher Scientific, MA, USA) and reconstituted in 200 µl of 0.1% FA in UPW.
   4. **Liquid chromatography (LC) conditions**
      A Nexera X2 high-performance liquid chromatography (HPLC) system (Shimadzu, Kyoto, Japan) equipped with a PFP column (2.1 mm × 150 mm, 4.6 µm particle size, GL Sciences) was used for online LC-MS/MS coupled with an LCMS-8050 mass spectrometer (Shimadzu). Solvent A was 0.1% FA in UPW, and solvent B was 0.1% FA in acetonitrile. The gradient program was: 0 min, 0% B; 0–10 min, 0–20% B; 10–15 min, 95% B; 15–20 min, 0% B. The flow rate was 0.4 ml/min, and the column oven was set at 40°C. Samples were kept at 4°C in the autosampler until a 10 µl injection.
   5. **Mass spectrometry (MS) conditions**
      MS/MS analysis was performed using an LCMS-8050 triple-quadrupole instrument with electrospray ionization (ESI) in positive ion mode. The parameters were: electrospray voltage, 0.7 kV; ESI interface temperature, 300°C; desolvation temperature, 526°C; desolvation line temperature, 250°C; heat block temperature, 400°C. Nitrogen gas flow rates for nebulizing, heating, and drying were set at 3, 10, and 10 L/min, respectively. High-purity argon gas at 270 kPa was used for collision-induced dissociation. Multiple reaction monitoring (MRM) transitions were m/z 112.10 → 95.15 for histamine and m/z 116.00 → 99.15 for d4-histamine. MS conditions for each analyte were optimized using automated MRM optimization in LabSolutions software (Shimadzu). The MRM dwell time was 100 ms. Histamine concentrations were quantified using LabSolutions with the internal standard.

**Abbreviations**
IS: Internal Standard
UPW: Ultrapure Water
FA: Formic Acid
HPLC: High-Performance Liquid Chromatography
ESI: Electrospray Ionization
MRM: Multiple Reaction Monitoring
